# Pregnant women’s knowledge, attitudes and perceptions toward umbilical cord blood banking

**DOI:** 10.1186/s41043-025-00837-y

**Published:** 2025-04-28

**Authors:** Sarah Sadi, Ahmad Darwish, Hesham Abdel-Hady, Doaa Alemam, Sohier Yahia, Nora El-Tantawy

**Affiliations:** 1 Family medicine specialist, Ministry of Health, Mansoura, Egypt; 2 Department of Pediatrics, Faculty of Medicine, Mansoura University, Mansoura, Egypt; 3 Faculty of Medicine, Mansoura Research Center for Cord Stem Cells (MARC-CSC), Mansoura University, Mansoura, Egypt; 4 Department of Public health and Community Medicine, Faculty of Medicine, Mansoura University, Mansoura, Egypt; 5 Department of Public health and Community Medicine, Faculty of Medicine, Horus university, Damietta, Egypt; 6 Department of Parasitology, Faculty of Medicine, Mansoura University, Mansoura, Egypt; 7 Department of Public Health, Faculty of Applied Medical Sciences, Al-Baha University, Al-Baha, Saudi Arabia

**Keywords:** Knowledge, Attitude, Perceptions, Umbilical cord blood, Banking, Mothers

## Abstract

**Background:**

Stem cell banking and donation hold great potential for the management of many diseases. The collection and banking of umbilical cord stem cells are essential for advancing and promoting stem cell-based therapy. At the heart of this promising field lies a crucial factor: the knowledge, attitudes, and perceptions of mothers toward stem cell donation and banking. Assessing how mothers perceive and engage with the concept of donating stem cells is pivotal in enhancing donation rates and ultimately saving lives. This study aims to assess expectant mothers’ knowledge, attitudes and perceptions with respect to umbilical cord blood (UCB) donation and banking.

**Methods:**

This cross-sectional study was conducted among pregnant women attending antenatal care clinics. The semistructured questionnaire includes sociodemographics data and information about mothers’ knowledge, attitudes and perceptions about stem cell donation and banking. The Cronbach’s alpha coefficient approach was used to determine the reliability of the questionnaire.

**Results:**

The study enrolled a total of 508 pregnant women with a mean age of 29.88 ± 6.10 years. The total score of knowledge was 4.34±6.10, ranging from 0 to 20, and 81.9% of them had poor knowledge. There was a significant association between the educational levels of the participants and their knowledge scores. The total attitude score was 11.45±2.60, and 49.6% of them had positive attitudes. The total score of the participants’ perceptions of cord blood banking was 8.13±9.84, and only 13.4% had high perceptions, while most of them (86.6%) had low perceptions. Mothers’ knowledge was strongly positively correlated with their attitudes (*r* =.260, *P* =.0031) and perceptions (*r* =.249, *P* =.0047) about cord blood banking.

**Conclusion:**

Most pregnant participants had poor knowledge, neutral attitudes and low perceptions about umbilical cord blood banking. Knowledge and attitudes are significantly correlated. Hence, implementing educational programs to increase knowledge and awareness of cord blood banking is crucial to empower mothers to share a pivotal role in the noble endeavor of saving lives through stem cell donation.

**Supplementary Information:**

The online version contains supplementary material available at 10.1186/s41043-025-00837-y.

## Introduction

Blood that remains in the placenta and attached to the umbilical cord after delivery is termed umbilical cord blood (UCB). There are specific differences in the blood composition between cord blood and whole blood. For example, cord blood has a greater proportion of immature T cells, a lower absolute number of T cells, and more natural killer cells [1]. However, the main reason for curiosity in cord blood is that it also contains a variety of stem and progenitor cells, primarily hematopoietic stem cells. Cord blood does contain certain non hematopoietic stem cell types, such as mesenchymal stem cells, but in considerably fewer quantities than adult bone marrow does. Compared with pluripotent embryonic stem cells, cord blood stem cells are more multipotent [2].

Any treatment for a disease or medical disorder that essentially utilizes any type of viable human stem cells, such as adult stem cells for autologous and allogeneic therapies and embryonic stem cells, is termed stem cell-based therapy. As stem cells may differentiate into the specific cell types required to promote the healing of damaged tissues, they offer the ideal option when tissue and organ transplantation is indicated [3]. However, because stem cell-based therapies are complicated, scientists frequently search for reliable, safe and conveniently accessible sources of stem cells that can differentiate into different lineages [4, 5]. Since then, cord blood banking has progressed to the point where approximately 800,000 units are stored in public banks and over 4 million units are preserved in private banks worldwide. More than 40,000 UCB harvests have been performed on both adults and children to treat a variety of diseases, including neurological, hematologic, immunologic, metabolic, and neoplastic disorders [6].

Women and their family members must be informed about UCB donation and its possible applications during pregnancy because this can raise awareness and encourage donations, which will lead to more cord blood units being available for transplantation. Patients with a family history of particular diseases may appreciate education in managing a variety of illnesses [7].

In the realm of modern healthcare, stem cell banking has become evident as a revolutionary technology with the potential to transform medical management and therapies. As mothers play a pivotal role in decision-making regarding their children’s health, understanding their knowledge, attitudes, and awareness toward stem cell banking becomes imperative. Studies have shown that maternal education and awareness significantly influence the choices made regarding the storage of newborn stem cells for potential future medical use [8]. Therefore, investigating the depth of mothers’ understanding of stem cell banking and their attitudes toward this practice is crucial in elucidating their preferences and concerns [9].

Maternal knowledge about stem cell banking can vary widely and can be affected by factors such as education level, socioeconomic status, and information source exposure [10]. Research has indicated that mothers who are well informed about the benefits of stem cell banking are more likely to opt to store their child’s stem cells [11]. Moreover, mothers’ attitudes toward stem cell banking can shape their choices regarding the investment in this medical insurance for their children’s future well-being. Positive attitudes toward the prospect of stem cells in regenerative medicine can lead to a higher acceptance rate among mothers for banking their newborn stem cells [12]. In contrast, concerns about the cost, efficacy, and ethical considerations surrounding stem cell banking may deter some mothers from pursuing this option [13]. Understanding these varying attitudes is vital for healthcare providers and policymakers to tailor their information and services effectively to meet the needs and perceptions of mothers contemplating stem cell banking [8].

Behavioral frameworks like the Health Belief Model (HBM) can be used to explain how pregnant women’s knowledge, attitudes, and perceptions impact their decision-making process regarding cord blood banking. The HBM states that people’s beliefs of their vulnerability to health risks, the seriousness of possible consequences, the advantages of taking action, the obstacles to taking action, the cues to take action, and their level of self-efficacy all influence their participation in health-related behaviors [14]. Pregnant women’s knowledge about the medical benefits, procedural aspects, and future potential of cord blood banking directly impacts their perceived benefits and barriers — for example, understanding that cord blood can treat life-threatening conditions may enhance their motivation to bank, while a lack of knowledge may raise doubts and perceived obstacles [15]. Similarly, attitudes are shaped by how women perceive the severity of diseases treatable by cord blood and their susceptibility to such conditions, influencing their willingness to participate [16]. Furthermore, perceptions, including the belief in the future utility of stored cord blood and trust in the healthcare system to manage the process, align with the HBM constructs of self-efficacy and cues to action [17]. For instance, healthcare provider recommendations and accessible educational materials can act as critical cues prompting women to consider banking. Thus, understanding pregnant women’s knowledge, attitudes, and perceptions through the lens of HBM not only explains their decision-making patterns but also highlights the need for targeted educational interventions to address gaps in knowledge, reshape attitudes, and manage perceptions effectively.

Awareness among mothers regarding the procedures, benefits, and limitations of stem cell banking is essential for making well-informed decisions. Studies have indicated that raising awareness through healthcare providers, antenatal classes, and informational materials can enhance mothers’ understanding of the advantages of storing umbilical cord blood stem cells. Enhancing awareness can dispel misconceptions and fears, empowering mothers to consider stem cell banking a valuable investment in their children’s health [18]. Therefore, efforts to increase awareness and knowledge among mothers about stem cell banking are crucial for promoting informed decision-making and ensuring the best possible healthcare outcomes for future generations. Due to the scarcity of data of data from Egypt and similar cultural contexts regarding the knowledge, attitudes and perceptions level of pregnant women toward cord blood banking, the aim of this research was to assess the current status of pregnant women toward umbilical cord blood banking in terms of their knowledge, attitudes and perceptions toward cord blood banking in Egypt to help optimize efforts and improve education among pregnant women, which might be employed in the establishment of appropriate policies and procedures for cord blood banking in public and private settings.

## Subjects and methods

### Study design and participants

This cross-sectional questionnaire-based study was conducted from January 2021 to July 2023 and targeted pregnant women attending the antenatal care clinic of Mansoura University Hospital, Egypt. The Institutional Review Board of Mansoura University’s Faculty of Medicine issued its approval for the study (code number: MS.21.5.1517). Direct face-to-face interviews were conducted with the participants to gather data. Each participating woman provided her verbal consent after being informed about the study’s purpose. Women were informed that participation in the study was completely optional and that they were free to stop answering the questionnaire at any moment. All publications and presentations included only aggregate data, and all personal data in the response form were kept confidential throughout the study.

### Sample size calculation

Sample size calculation was performed via this formula $\:\:\:\text{n}=\:\frac{{Z}^{2}P\:\left(1-P\right)}{{d}^{2}}$ [19], where Z is 1.96 for a 95% confidence interval, *P* is the expected prevalence of poor knowledge among pregnant women, which is 74% according to Pandey et al. [20], and d is precision (margin of error) (0.05). Therefore, the calculated sample size was at least 127 participants.

### Study tool

The questionnaire used consists of four parts. The first part concerns sociodemographic data, including women’s age, educational status, parity, trimester, history of chronic diseases, and health-related data. The other three parts assess knowledge, attitudes and mothers’ perceptions about stem cell donation. A review of survey questions that evaluated participants’ knowledge, attitudes and willingness to use stem cells was performed. The survey tool for this study included a set of questions that were developed on the basis of the review [20–22]. This survey’s items were evaluated by field experts to verify the relevance of the questions considering the objectives of the study. With the assistance of a bilingual researcher, the forward and backward translation approach was employed to translate the questionnaire from English into Arabic. A panel of experts who were consulted ensured the questionnaire’s content validity and amended the questionnaire on the basis of their feedback.

Twenty pregnant women who were not included in the full-scale study were assigned at random to participate in a pilot study of the final Arabic version of the questionnaire. They were recruited from the Antenatal Care Clinics, Gynecology and Obstetrics Department, Mansoura University Hospital, Egypt.

A pilot test was conducted to determine the validity and clarity of the study questionnaire, estimate how long it would take to complete, and calculate the percentage of non respondents or missing data.

To render questions simpler to comprehend and interpret, the questionnaires were modified to accommodate cultural differences. Small changes such as clarifying the phrasing or adding examples or contextual information would help certain questions. Data were gathered through in-person interviews with each woman by the researcher. Each interview with a pregnant woman lasted between 20 and 30 min.

The internal reliability of the questions was investigated. The Cronbach’s alpha test was employed to evaluate the internal consistency of the questionnaire. The knowledge domain yielded a Cronbach’s alpha of 0.917 (strong), the attitude domain had a lower alpha of 0.550 (satisfactory), and the mothers’ perceptions domain yielded a value of 0.944 (excellent). The relatively low Cronbach’s alpha (α = 0.55) for the attitude domain may be attributed to several factors. First, the attitude domain included a limited number of items, which can reduce internal consistency. Second, the items in this domain were designed to capture diverse aspects of attitudes toward cord blood banking, including both positive and negative views. This conceptual diversity may have reduced the inter-item correlations and, consequently, the overall alpha value. We suggest to refine and expand the attitude items to improve internal consistency in future studies to solve this imitation.

The knowledge domain consisted of 14 questions. The number of correct answers is 2, and the number of incorrect answers is zero. The total knowledge score ranged from zero to 28. Three categories were classified when the total score was considered: poor knowledge score, ranging from 0 to 10; average knowledge score, 11–18; and good knowledge score, 19–28. The mother’s attitude domain is composed of 10 items, and a three-point Likert scale is used to award a score to each item. Each one received a score of (Zero) for disagreement [1], for uncertainty, and [2] for agreement. The total score of attitudes was divided into two categories and ranged from zero to 20: positive attitudes ≥ 60% (scores of 12–20) and negative attitudes < 60% (scores of 0–11). The mothers’ perceptions about stem cell donation domain is composed of 10 items, and every item of the perception scale is given a score on a five-point Likert scale. Every statement was scored as [4] excellent [3], very good [2], good [1], accepted or (zero) weak. The total score of perception ranged from zero to 40 and was divided into 2 categories: high perception ≥ 60% (scores of 24–40) and low perception < 60% (scores of zero–23). Participants’ responses with missing values were excluded.

### Statistical analysis

All of the data were collected, tabulated, and statistically analyzed via SPSS version 22, a statistical program for special science (SPSS Inc. Chicago, IL, U.S.A.). The quantitative data are expressed as the median and range for non-parametric data and the mean ± standard deviation (SD) for parametric data. Frequencies and relative percentages were used to express the qualitative data. The difference between qualitative variables was determined via Fisher’s exact test and the chi square test (χ^2^). Every statistical comparison had a two-tailed p value significance threshold. A *P* value $\:\le\:\:$0.05 indicated significance.

## Results

A total of five hundred and eight pregnant women participated in the research. The mean age of the women was 29.88 ± 6.10 years, 29.9% of them were aged 31–35 years, 52% had a secondary education level, 59.8% were housewives, 63% lived in rural areas, 74.8% were multipara, 39.4% were in their 2nd trimester, and 42.5% had no history of chronic or blood diseases.

### Mothers’ knowledge about cord blood banking

With respect to mothers’ knowledge about cord blood banking, the total knowledge score was 28 points, with median (Min.– Max.) was 2 (0–20). The knowledge level distributions were 81.9%, 15%, and 3.1% for poor, average and good knowledge scores, respectively. Table (1) shows the distribution of mothers according to their knowledge of UCB banking. There was a significant association between the educational levels of the enrolled mothers and their knowledge score (*P* =.045), whereas there were no statistically significant associations between mothers’ degree of knowledge and their age (*P =*.526), occupation (*P* =.706), residence (*P* =.277), parity (*P* =.106), trimester (*P* =.118) or family history of disease (*P* =.414), as shown in Table (2).

**Table 1:** Distribution of pregnant women according to their knowledge items about UCB banking

Items	Correct	Incorrect
1. Definition of umbilical cord blood	368 (72.4%)	140 (27.6%)
2. Awareness about the person/s that donate cord blood	64 (12.6%)	444 (87.4%)
3. Purposes of umbilical cord blood collection	92 (18.1%)	416 (81.9%)
4. Contraindications of cord blood collection	0 (0%)	508 (100%)
5. Appropriate time for collecting cord blood	52 (10.2%)	456 (89.8%)
6. Maximum duration of cord blood storage	16 (3.1%)	492 (96.9%)
7. Illnesses that can be managed by cord blood	92 (18.1%)	416 (81.9%)
8. Stem cells definition	64 (12.6%)	444 (87.4%)
9. Umbilical cord composed of supernatural tissue or nerve or skin cells	36 (7.1%)	592 (92.9%)
10. Umbilical cord blood can be collected during the normal delivery or Caesarean section	108 (21.3%)	400 (78.7%)
11. Only the same child could benefit from umbilical cord blood	16 (3.1%)	492 (96.9%)
12. Cord umbilical tissue contains stem cells	36 (7.1%)	472 (92.9%)
13. The main rationale for banking umbilical cord blood	108 (21.3%)	400 (78.7%)
14. Being aware of our country’s cord blood banking	56 (11.1%)	452 (88.9%)

**Table 2:** Association between mother’s knowledge score level and their clinical characteristics

Mothers’ characteristics	Total	Knowledge score			Sig. test
		Poor	Average	Good	
Mother age					X = 5.13
< 25 years	128 (100%)	100 (78.1%)	24 (18.7%)	4 (3.2%)	P =.526
25–30 years	136 (100%)	108 (79.4%)	20 (14.7%)	4 (5.9%)	
31–35 years	152 (100%)	140 (92.1%)	12 (7.9%)	0 (00%)	
> 35 years	92 (100%)	68 (73.9%)	20 (21.7%)	1 (4.3%)	
Educational levels					X = 12.91
Illiterate	24 (100%)	20 (83.3%)	4 (16.7%)	0 (00%)	P =.045
Primary education	76 (100%)	68 (89.5%)	8 (10.5%)	0 (00%)	
Secondary education	264 (100%)	224 (84.9%)	36 (13.6%)	4 (1.5%)	
University education	144 (100%)	104 (72.2%)	28 (19.4%)	12 (8.3%)	
Occupation					X =.69
`Housewife	304 (100%)	256 (84.2%)	40 (13.2%)	8 (2.6%)	P =.706
Working	204 (100%)	160 (78.4%)	36 (17.6%)	8 (3.9%)	
Residence					X = 2.56
Urban	188 (100%)	148 (78.7%)	28 (14.9%)	12 (6.4%)	P =.277
Rural	320 (100%)	268 (83.7%)	48 (15%)	4 (1.2%)	
Parity Primipara Multipara	128 (100%) 380 (100%)	96 (75%) 320 (84.2%)	32 (25%) 44 (11.6%)	0 (00%) 16 (4.2%)	X = 4.47 P =.106
Trimester					X = 7.35
1st trimester	132 (100%)	116 (87.9%)	12 (9.1%)	4 (3.0%)	P =.118
2nd trimester	200 (100%)	176 (88%)	24 (12%)	0 (00%)	
3rd trimester	176 (100%)	124 (70.5%)	40 (22.7%)	12 (6.8%)	
Family history of diseases					X = 3.94
No history of diseases	216 (100%)	172 (79.6%)	40 (18.5%)	4 (1.8%)	P =.414
Chronic diseases	196 (100%)	156 (79.6%)	28 (14.3%)	12 (6.1%)	
Blood diseases	96 (100%)	88 (91.7%)	8 (8.3%)	0 (00%)	
Total	508 (100%)	416 (81.9%)	76 (15%)	16 (3.1%)	

*P* <.05 is significant

### Mothers’ attitudes toward cord blood banking

With respect to mothers’ attitudes toward cord blood banking, 83.5% of the enrolled mothers agreed with the statement that stored umbilical cord blood should be available to everyone, 83.5% of the respondents agreed that my baby was not harmed by cord blood sampling, 87.4% agreed that the baby’s cord blood can be applied to various goals, 77.2% preferred to preserve the baby’s cord blood in private banks in preference to public banks despite low perceptions, and 75.6% agreed to donate my baby cord blood, as shown in Table (3). The mean attitude score was 11.45 ± 2.60, ranging from 4 to 18. A total of 49.6% of the participants had positive attitudes, whereas 50.4% had negative attitudes. There were significant associations between the attitude scores of the enrolled mothers and their age (*P* =.001), educational level (*P* <.0001), occupation (*P* <.001), residence (*P* <.001), parity (*P* <.001) and trimester (*P* <.001). However, there was no significant association between the attitudes of the enrolled mothers and their family history of disease (*P* =.220), as shown in Table (4).

**Table 3:** Distribution of mothers according to their attitude items regarding UCB banking

Item	Agree	Uncertain	Disagree
1. It is more reliable to use my baby’s own cord blood rather than someone else’s.	44 (8.7%)	188 (37%)	276 (54.3%)
2. Only my own family should receive my baby’s cord blood.	104 (20.5%)	92 (18.1%)	312 (61.4%)
3. I am willing to preserve umbilical cord blood for my unborn child if the cost is affordable.	160 (31.5%)	88 (17.3%)	260 (51.2%)
4. Everyone should have access to the stored umbilical cord blood if needed.	424 (83.5%)	48 (9.4%)	36 (7.1%)
5. There was no harm to my baby from the cord blood collection.	424 (83.5%)	48 (9.4%)	36 (7.1%)
6. Donating a cord blood sample is not necessary.	36 (7.1%)	96 (18.9%)	376 (74%)
7. There are various uses for baby cord blood.	444 (87.4%)	24 (4.7%)	40 (7.9%)
8. Rather than storing my baby’s cord blood in public banks, I prefer to keep it in private ones.	432 (77.2%)	4 (0.8%)	112 (22%)
9. Cord blood storage services are only available to babies born in private hospitals.	76 (15%)	148 (29.1%)	284 (55.9%)
10. I’d agree with donating my baby cord blood.	384 (75.6%)	48 (9.4%)	76 (15%)

**Table 4:** Association between mothers’ characteristics and their attitude level about UCB banking

Mothers’ characteristics	Total	Attitude score		Significance
		Positive attitude	Negative attitude	
Mother age in years				X = 22.28
< 25	128 (100%)	92 (72.9%)	36 (28.1%)	P =.001
25–30	136 (100%)	76 (55.9%)	60 (44.1%)	
31–35	152 (100%)	76 (50.0%)	76 (50.0%)	
> 35	92 (100%)	8 (8.7%)	84 (91.3%)	
Educational level				X = 38.18
Illiterate	24 (100%)	0 (0%)	24 (100%)	P <.001
Primary education	76 (100%)	12 (15.8%)	64 (84.2%)	
Secondary education	264 (100%)	112 (42.4%)	152 (57.6%)	
University education	144 (100%)	128 (88.9%)	16 (11.1%)	
Occupation				X = 64.60
Housewife	304 (100%)	60 (19.7%)	244 (80.3%)	P <.001
Working	204 (100%)	192 (94.1%)	12 (5.9%)	
Residence				X = 31.15
Urban	188 (100%)	156 (82.9%)	32 (17.1%)	P <.001
Rural	320 (100%)	96 (30.0%)	224 (70.0%)	
Parity				X = 22.588
Primipara	128 (100%)	112 (87.5%)	16 (12.5%)	P <.0001
Multipara	380 (100%)	140 (36.8%)	240 (63.2%)	
Trimester				X = 29.19
1st trimester	132 (100%)	20 (15.2%)	112 (84.8%)	P <.001
2nd trimester	200 (100%)	96 (48.0%)	104 (52.0%)	
3rd trimester	176 (100%)	136 (77.3%)	40 (22.7%)	
Family history of diseases				X = 3.02
No history of diseases	216 (100%)	88 (40.7%)	128 (59.3%)	P =.220
Chronic diseases	196 (100%)	108 (55.1%)	88 (44.9%)	
Blood diseases	96 (100%)	56 (58.3%)	40 (41.7%)	
Total	508 (100%)	252 (49.6%)	256 (50.4%)	

*P* <.05 is significant

### Mothers’ perceptions about cord blood banking

The perceptions of mothers and their satisfaction with UCB banking are presented in Table (5). The mean perceptions score among the 508 enrolled mothers was 8.13 ± 9.84, ranging from 0 to 40. Only 13.4% have high perceptions, while most (86.6%) of them have low perceptions. There were significant associations between mothers’ perceptions scores and age (*P* =.009), educational level (*P* =.036), residence (*P* <.001) and family history of disease (*P* <.001). There were no significant associations between mothers’ perception scores and occupation, parity or trimester (*P* <.05), as shown in Table (6).

**Table 5:** Distribution of mothers according to their expectation and their satisfaction items about UCB banking

Items	Weak	Accepted	Good	Very good	Excellent
1. UCB can be stored for use in the future for up to 20 years.	116 (22.8%)	0	144 (28.3%)	140 (27.6%)	108 (21.3%)
2. Cancer can be treated using UCB.	392 (77.2%)	8 (1.6%)	80 (15.7%)	0	28 (5.5%)
3. UCB assists in managing chronic illnesses like diabetes and hypertension	252 (49.6%)	20 (3.9%)	148 (29.1%)	56 (11.0%)	32 (6.3%)
4-UCB will be useful in future research to develop regenerative medicine approaches.	444 (87.4%)	0	40 (7.9%)	0	24 (4.7%)
5. I worry that stem cell transplantation might render it easier for people to kill for the benefit of others.	200 (39.4%)	40 (15.7%)	76 (15.0%)	24 (4.7%)	128 (25.2%)
6. It is not a waste of time or effort to store umbilical cord blood.	148 (29.1%)	120 (23.6%)	72 (14.2%)	24 (4.7%)	144 (28.3%)
7. Stem cell transplantation needs to be widespread.	172 (33.9%)	0	72 (14.2%)	120 (23.6%)	144 (28.3%)
8. Stem cell transplantation can save lives.	28 (5.5%)	0	72 (15.0%)	24 (4.7%)	380 (74.8%)
9. Stem cell therapy and cord blood harvesting are accepted in Islam.	28 (5.5%)	0	72 (14.2%)	24 (4.7%)	384 (75.6%)
10. UCB units can still be used for transplantation years after they are first stored.	72 (14.2%)	0	64 (12.6%)	60 (11.8%)	312 (61.4%)

**Table 6:** Association between mothers’ characteristics and their expectation level about UCB banking

Mothers’ characteristics	Total	Expectation score		Significance
		Low	High	
Mother age in years				X = 16.41
< 25	128 (100%)	120 (93.8%)	8 (6.2%)	P =.009
25–30	136 (100%)	124 (91.2%)	12 (8.8%)	
31–35	152 (100%)	104 (68.4%)	48 (31.6%)	
> 35	92 (100%)	92 (100%)	0 (0%)	
Educational level				X = 10.24
Illiterate	24 (100%)	24 (100%)	0 (0%)	P =.036
Primary education	76 (100%)	68 (89.5%)	8 (10.8%)	
Secondary education	264 (100%)	244 (92.4%)	20 (7.6%)	
University education	144 (100%)	104 (72.2%)	40 (27.8%)	
Occupation				X = 3.813
Housewife	304 (100%)	280 (92.1%)	24 (7.9%)	P =.051
Working	204 (100%)	160 (78.4%)	44 (21.6%)	
Residence				X = 19.63
Urban	188 (100%)	128 (68.1%)	60 (31.9%)	P <.001
Rural	320 (100%)	312 (97.5%)	8 (2.5%)	
Parity				X = 3.72
Primipara	128 (100%)	96 (75.0%)	32 (25.0%)	P =.053
Multipara	380 (100%)	344 (90.5%)	36 (9.5%)	
Trimester				X = 1.48
1st trimester	132 (100%)	10 (90.9%)	12 (9.1%)	P =.477
2nd trimester	200 (100%)	176 (88.0%)	24 (12.0%)	
3rd trimester	176 (100%)	144 (81.8%)	32 (18.2%)	
Family history of diseases				X = 27.12
No history of diseases	216 (100%)	200 (92.6%)	16 (7.4%)	P <.001
Chronic diseases	196 (100%)	188 (95.9%)	8 (4.1%)	
Blood diseases	108 (100%)	52 (54.2%)	44 (45.8%)	
Total	508 (100%)	440 (86.6%)	68 (13.4%)	

*P* <.05 is significant

### Mothers’ attitudes, perceptions, and knowledge of UCB banking correlations

**Fig. 1:**
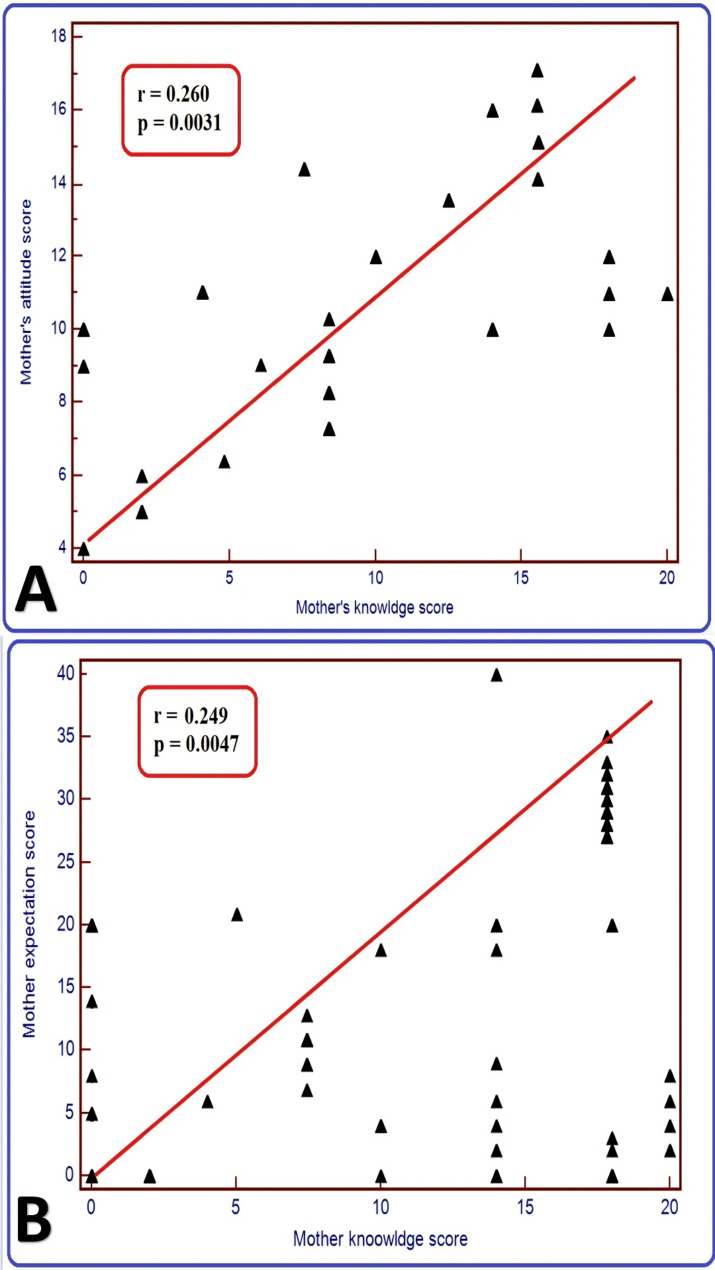
Scatter plot chart showing the correlation between (A) mothers’ knowledge and their attitude about UCB banking and (B) mothers’ knowledge and expectations about UCB banking

Mothers’ attitudes and their level of knowledge were weakly positively correlated (*r* =.260, *P* =.0031) and perceptions (*r* =.249, *P* =.0047) about UCB banking. As the knowledge score of the studied women increased, their attitudes and perceptions increased, as shown in Figure (1).

## Discussion

Pregnant women need to be granted access to the data they need to make a knowledgeable decision and be mindful of the choices for cord blood that they have available. According to publications, women in Egypt are not properly educated regarding cord blood banking and donation and are somewhat familiar with the quality of the data available to them or their source [18]. With respect to mothers’ knowledge regarding cord blood banking in this study, the total knowledge score was 28, with a mean of (4.34 ± 6.10). The knowledge level distributions were 81.9%, 15%, and 3.1% for poor, average and good knowledge scores, respectively. There was a significant association between the educational levels of the enrolled mothers and their knowledge score (*P* =.045), whereas there were no statistically significant associations between mothers’ knowledge level and their occupation, age, residence, parity, trimester or family history of disease. Our findings revealed that 72.4% of the enrolled mothers defined the umbilical cord correctly: 18.1% of them were aware of the purpose of umbilical cord blood collection, 18.1% were aware of the importance of UCB for illnesses that can be managed by cord blood, 21.3% of them were aware that UCB can be collected during normal delivery or Cesarean section, 21.3% of them were aware of the main reason for umbilical cord blood banking, and most of them were incorrectly aware of other items with knowledge scores. 100% of the enrolled mothers incorrectly answered about contraindications for cord blood collection. A total of 96.9% ignore the maximum duration of cord blood storage, and 96.9% of the UCB should be used for the same child only.

Similarly, Awad et al. [12] noted that pregnant women (78.5%) who were aware of the definition of the umbilical cord had low knowledge levels regarding other parameters related to the collection and processing of cord blood stem cells and their donation and banking. This result is in line with that of Subramaniam et al. [23], who investigated pregnant women’s knowledge and attitudes toward cord blood banking in Malaysia. They reported that only 11.5% of participants had a basic understanding of cord blood banks, only 18% were aware of the main purpose of UCB, and over 90% were unaware of the procedures involved in collecting and storing cord blood. Approximately 80% of them were unaware of the idea of public cord blood banking.

According to a 2017 study by Habib et al., [24] just 7% of the women understood what cord blood was, only 17% understood what stem cells were, just 8.6% understood the significance of obtaining stem cells from the umbilical cord, and only 8.3% understood the applications of stem cells. These previous results are consistent with those of a study investigating public awareness of cord blood banking conducted in Saudi Arabia by Jawdat et al. [25], which also reported similar results.

With respect to mothers’ attitudes regarding UCB banking, 83.5% of the enrolled mothers agreed that stored umbilical cord blood should be available to everyone, 83.5% agreed that there is no harm to the baby during cord blood collection, 87.4% agreed that cord blood can be utilized for many goals, 77.2% wanted to preserve her baby’s cord blood in private banks in preference to public ones, and 75.6% would accept baby cord blood donation. Cultural factors play a significant role in shaping preferences for stem cell banking in different regions. In Egypt, there is a strong preference for private stem cell banks due to a combination of factors such as the political and funding instabilities in the country that slow the public banks development, limited regulation, and the perception that private services offer higher quality and more personalized care [26]. In contrast, India has seen greater acceptance of public stem cell banks, driven by government initiatives aimed at making stem cell services more affordable and accessible to the broader population [27]. Cultural attitudes toward healthcare, trust in public versus private services, and socio-economic conditions influence these regional differences in stem cell banking preferences. A total of 49.6% of the enrolled mothers had positive attitudes, whereas 50.4% had negative attitudes, with a total attitude score of 11.45 ± 2.60. Consistent with our research, Awad et al. [12] reported that approximately 49.5% of the participants in their study had a negative attitude toward the donation and banking of cord blood stem cells. Nonetheless, 50.5% of them had a positive attitude. Similarly, Habib et al. [24] reported that a sample’s attitude toward cord blood donation was favorable for 41% of participants, whereas 24% had a negative attitude. Only 15% of participants in a survey carried out by Pandey et al. [20] to assess the attitudes of possible donors from one of India’s largest UCB repositories were supportive, which was a lower finding. A significant percentage (55%) were uncertain about whether to bank UCB.

The findings of other studies reported different percentages. According to Aboushady et al. [22], 33% of participants had good attitudes, and 67% had negative attitudes. The findings of this study are consistent with those of Key Value Pair [26], who investigated pregnant women’s attitudes toward and knowledge of the preservation of umbilical cord blood. The majority of the samples responded negatively to blood and stem cell banking. Furthermore, Jawdat et al. [25] reported that approximately 2/3 of their study participants about public awareness of cord blood banking in Saudi Arabia lacked sufficient knowledge and had a negative attitude. Nearly all of the pregnant women in the Sahoo et al. [23] study—approximately 80.3%—had a positive attitude toward banking stem cells at public banks because they were less expensive than those at private banks. Additionally, Subramaniam et al. [23] noted that just 23% of their subjects had a favorable attitude toward UCB storage, whereas 22.4% declined, and a significant number (54.6%) were unsure. According to a study by Aboushady et al. [22], 33% of the study sample had a positive attitude regarding stem cells and cord blood banking, whereas 67% of the study population had a negative attitude.

From this point of view, the enrolled mothers had a total score of 8.13 ± 9.84 for perceptions about UCB banking. A total of 13.4% had high perceptions, while 86.6% had low perceptions. A total of 31.6% of mothers aged 31–35 years, 27.8% of those with a university education, 21.6% of working mothers, 31.9% of urban mothers, 25% of primipara, 18.2% of those in the third trimester and 45.8% of those with a history of blood diseases had high perception scores. There were powerful significant correlations between mothers’ perceptions scores and age (*P* =.009), educational level (*P* =.036), residence (*P* <.001) and family history of disease (*P* <.001). However, there were no significant associations between mothers’ perceptions and their occupation, parity or trimester (*P* >.05). Similar findings were reported by Aboushady et al. [22] and Awad et al. [12]. They reported low perception scores among their participants, with a significant association between mothers’ perceptions and satisfaction scores according to their age, educational level and residence.

The current study revealed weak positive correlations between mothers’ knowledge and their attitudes (*r* =.260, *P* =.0031) and perceptions (*r* =.249, *P* =.0047) about UCB banking. As the knowledge score of the studied women increased, their attitudes and perceptions increased. Similarly, studies have revealed a strong statistically significant positive relationship between the total knowledge score and the total attitude score of the enrolled mothers (*P* =.000) [12, 28, 29, 30]. Subramaniam et al. [23] reported that knowledge was strongly positively correlated with attitudes (*r* =.75; *P* <.001) and weakly but significantly correlated with perceptions to register with the Saudi Stem Cell Registry (*r* =.01; *P* <.01). The present findings may indicate that an improved awareness of the advantages and clinical importance of UCB will increase support for UCB donation and banking. This is supported by other previous studies showing that many participants had low perceptions for cord blood banking, primarily as a result of insufficient knowledge [28, 29, 31, 32]. The weak but statistically significant correlation (*r* =.26) between knowledge and attitudes suggests that while higher knowledge is associated with more positive attitudes, other factors may influence attitudes beyond knowledge alone. Personal beliefs, cultural values, emotional responses, trust in healthcare systems, and prior experiences may shape attitudes independently of factual knowledge. This indicates that increasing knowledge may not be sufficient to shift attitudes without also addressing these underlying factors. Similar findings in health behavior research show that knowledge often plays a limited role when social, emotional, and cultural factors are strong influencers.

The findings have important policy implications for Egypt’s healthcare infrastructure. The limited knowledge and mixed attitudes toward stem cell donation highlight the need for national awareness campaigns integrated into existing healthcare services. Policies should focus on establishing public stem cell banks regulated by clear guidelines to build trust and ensure equitable access. Integrating stem cell education into routine healthcare interactions, such as during prenatal care, can also raise awareness. Additionally, training healthcare providers to counsel patients about stem cell options would align with efforts to strengthen Egypt’s preventive and personalized healthcare approaches, supporting broader national health goals.

This study has some limitations. First, the reliance on self-reported data may introduce bias, as participants might overestimate or underestimate their knowledge and attitudes due to social desirability or recall errors. Second, the sample is relatively homogeneous in terms of demographic backgrounds, which may limit the generalization of the findings to more diverse populations. Future research should include more diverse samples from different localities and consider using objective measures to validate self-reported responses.

## Conclusion

Our study revealed that pregnant women had little knowledge of cord blood stem cell banking and donation. There was a weak statistically significant correlation between participants’ total knowledge scores and their attitudes. This weak correlation suggest presence of other influencers as personal beliefs, cultural values, emotional responses, trust in healthcare systems, and prior experiences that may shape attitudes independently of factual knowledge. Therefore, culturally tailored interventions are essential to address specific beliefs and attitudes within different communities. One effective strategy is organizing community-based workshops to provide accurate information and address misconceptions about stem cell donation and banking to establish and expand the status of umbilical cord banking in Egypt.

## Electronic supplementary material

Below is the link to the electronic supplementary material.

Supplementary Material 1

## Data Availability

No datasets were generated or analysed during the current study.

## Abbreviations

UCB: Umbilical Cord blood
SD: Standard Deviation
HBM: Health Belief Model
