# Supplementary material for: Pregnant women’s knowledge, attitudes and perceptions toward umbilical cord blood banking
Source: J Health Popul Nutr. 2025 Apr 28;44:140. doi: 10.1186/s41043-025-00837-y (PMC12038976; doi:10.1186/s41043-025-00837-y)
Supplement: Supplementary file 1 — Supplementary Material 1 [file 41043_2025_837_MOESM1_ESM.docx]

Table 1: Distribution of pregnant women according to their knowledge items about UCB banking

| **Items** | **Correct**  **Number (percent)** | **Incorrect**  **Number (percent)** |
| --- | --- | --- |
| 1. Definition of umbilical cord blood | 368 (72.4) | 140 (27.6) |
| 2. Awareness about the person/s that donate cord blood | 64 (12.6) | 444 (87.4) |
| 3. Purposes of umbilical cord blood collection | 92 (18.1) | 416 (81.9) |
| 4. Contraindications of cord blood collection | 0 (0) | 508 (100) |
| 5. Appropriate time for collecting cord blood | 52 (10.2) | 456 (89.8) |
| 6. Maximum duration of cord blood storage | 16 (3.1) | 492 (96.9) |
| 7. Illnesses that can be managed by cord blood | 92 (18.1) | 416 (81.9) |
| 8. Stem cells definition | 64 (12.6) | 444 (87.4) |
| 9. Umbilical cord composed of supernatural tissue or nerve or skin cells | 36 (7.1) | 592 (92.9) |
| 1. Umbilical cord blood can be collected during the normal delivery or Caesarean section | 108 (21.3) | 400 (78.7) |
| 11.Only the same child could benefit from umbilical cord blood | 16 (3.1) | 492 (96.9) |
| 12. Cord umbilical tissue contains stem cells | 36 (7.1) | 472 (92.9) |
| 13. The main rationale for banking umbilical cord blood | 108 (21.3) | 400 (78.7) |
| 14. Being aware of our country's cord blood banking | 56 (11.1) | 452 (88.9) |

Table 2: Association between mother's knowledge score level and their clinical characteristics

| **Mothers’ characteristics** | **Total** | **Knowledge score** | | | **Sig. test** |
| --- | --- | --- | --- | --- | --- |
|  |  | **Poor** | **Average** | **Good** |  |
|  | **Number (Percent)** | | | |  |
| Mother age  <25 years  25-30 years  31-35 years  >35 years | 128 (100)  136 (100)  152 (100)  92 (100) | 100 (78.1)  108 (79.4)  140 (92.1)  68 (73.9) | 24 (18.7)  20 (14.7)  12 (7.9)  20 (21.7) | 4 (3.2)  4 (5.9)  0 (00)  1 (4.3) | X=5.13 *P*=0.526 |
| Educational levels  Illiterate  Primary education  Secondary education  University education | 24 (100)  76 (100)  264 (100)  144 (100) | 20 (83.3)  68 (89.5)  224 (84.9)  104 (72.2) | 4 (16.7)  8 (10.5)  36 (13.6)  28 (19.4) | 0 (00)  0 (00)  4 (1.5)  12 (8.3) | X=12.91 *P*=0.045 |
| Occupation  Housewife  Working | 304 (100)  204 (100) | 256 (84.2)  160 (78.4) | 40 (13.2)  36 (17.6) | 8 (2.6)  8 (3.9) | X=.69  *P*=0.706 |
| Residence  Urban  Rural | 188 (100)  320 (100) | 148 (78.7)  268 (83.7) | 28 (14.9)  48 (15) | 12 (6.4)  4 (1.2) | X=2.56 *P*=0.277 |
| Parity  Primipara  Multipara | 128 (100)  380 (100) | 96 (75)  320 (84.2) | 32 (25)  44 (11.6) | 0 (00)  16 (4.2) | X=4.47 *P*=0.106 |
| Trimester  1st trimester  2nd trimester  3rd trimester | 132 (100)  200 (100)  176 (100) | 116 (87.9)  176 (88)  124 (70.5) | 12 (9.1)  24 (12)  40 (22.7) | 4 (3.0)  0 (00)  12 (6.8) | X=7.35 *P*=0.118 |
| Family history of diseases  No history of diseases  Chronic diseases  Blood diseases | 216 (100)  196 (100)  96 (100) | 172 (79.6)  156 (79.6)  88 (91.7) | 40 (18.5)  28 (14.3)  8 (8.3) | 4 (1.8)  12 (6.1)  0 (00) | X=3.94 *P*=0.414 |
| Total | 508 (100) | 416 (81.9) | 76 (15) | 16 (3.1) |  |

*P*<0.05 is significant

Table 3: Distribution of mothers according to their attitude items regarding UCB banking

| **Item** | **Agree** | **Uncertain** | **Disagree** |
| --- | --- | --- | --- |
|  | **Number (Percent)** | | |
| 1. It is more reliable to use my baby's own cord blood rather than someone else's. | 44 (8.7) | 188 (37) | 276 (54.3) |
| 2. Only my own family should receive my baby's cord blood. | 104 (20.5) | 92 (18.1) | 312 (61.4) |
| 3. I am willing to preserve umbilical cord blood for my unborn child if the cost is affordable. | 160 (31.5) | 88 (17.3) | 260 (51.2) |
| 4. Everyone should have access to the stored umbilical cord blood if needed. | 424 (83.5) | 48 (9.4) | 36 (7.1) |
| 5. There was no harm to my baby from the cord blood collection. | 424 (83.5) | 48 (9.4) | 36 (7.1) |
| 6. Donating a cord blood sample is not necessary. | 36 (7.1) | 96 (18.9) | 376 (74) |
| 7. There are various uses for baby cord blood. | 444 (87.4) | 24 (4.7) | 40 (7.9) |
| 8. Rather than storing my baby's cord blood in public banks, I prefer to keep it in private ones. | 432 (77.2) | 4 (0.8) | 112 (22) |
| 9. Cord blood storage services are only available to babies born in private hospitals. | 76 (15) | 148 (29.1) | 284 (55.9) |
| 10. I'd agree with donating my baby cord blood. | 384 (75.6) | 48 (9.4) | 76 (15) |

Table 4: Association between mothers’ characteristics and their attitude level about UCB banking

| **Mothers’ characteristics** | **Total** | **Attitude score** | | **Significance** |
| --- | --- | --- | --- | --- |
|  |  | **Positive**  **attitude** | **Negative attitude** |  |
|  | **Number (Percent)** | | |  |
| Mother age in years  <25  25-30  31-35  >35 | 128 (100)  136 (100)  152 (100)  92 (100) | 92 (72.9)  76 (55.9)  76 (50.0)  8 (8.7) | 36 (28.1)  60 (44.1)  76 (50.0)  84 (91.3) | X= 22.28  *P*= 0.001 |
| Educational level  Illiterate  Primary education  Secondary education  University education | 24 (100)  76 (100)  264 (100)  144 (100) | 0 (0)  12 (15.8)  112 (42.4)  128 (88.9) | 24 (100)  64 (84.2)  152 (57.6)  16 (11.1) | X=38.18  *P*<0.001 |
| Occupation  Housewife  Working | 304 (100)  204 (100) | 60 (19.7)  192 (94.1) | 244 (80.3)  12 (5.9) | X=64.60  *P*<0.001 |
| Residence  Urban  Rural | 188 (100)  320 (100) | 156 (82.9)  96 (30.0) | 32 (17.1)  224 (70.0) | X=31.15  *P*<0.001 |
| Parity  Primipara  Multipara | 128 (100)  380 (100) | 112 (87.5)  140 (36.8) | 16 (12.5)  240 (63.2) | X=22.588  *P*<0.0001 |
| Trimester  1st trimester  2nd trimester  3rd trimester | 132 (100)  200 (100)  176 (100) | 20 (15.2)  96 (48.0)  136 (77.3) | 112 (84.8)  104 (52.0)  40 (22.7) | X=29.19  *P*<0.001 |
| Family history of diseases  No history of diseases  Chronic diseases  Blood diseases | 216 (100)  196 (100)  96 (100) | 88 (40.7)  108 (55.1)  56 (58.3) | 128 (59.3)  88 (44.9)  40 (41.7) | X=3.02  *P*=0.220 |
| Total | 508 (100) | 252 (49.6) | 256 (50.4) |  |

*P*<0.05 is significant

Table 5: Distribution of mothers according to their expectation and their satisfaction items about UCB banking

| **Items** | **Weak** | **Accepted** | **Good** | **Very good** | **Excellent** |
| --- | --- | --- | --- | --- | --- |
|  | **Number (Percent)** | | | | |
| 1. UCB can be stored for use in the future for up to 20 years. | 116 (22.8) | 0 | 144 (28.3) | 140 (27.6) | 108 (21.3) |
| 2. Cancer can be treated using UCB. | 392 (77.2) | 8 (1.6) | 80 (15.7) | 0 | 28 (5.5) |
| 3. UCB assists in managing chronic illnesses like diabetes and hypertension | 252 (49.6) | 20 (3.9) | 148 (29.1) | 56 (11.0) | 32 (6.3) |
| 4-UCB will be useful in future research to develop regenerative medicine approaches. | 444 (87.4) | 0 | 40 (7.9) | 0 | 24 (4.7) |
| 5. I worry that stem cell transplantation might render it easier for people to kill for the benefit of others. | 200 (39.4) | 40 (15.7) | 76 (15.0) | 24 (4.7) | 128 (25.2) |
| 6. It is not a waste of time or effort to store umbilical cord blood. | 148 (29.1) | 120 (23.6) | 72 (14.2) | 24 (4.7) | 144 (28.3) |
| 7. Stem cell transplantation needs to be widespread. | 172 (33.9) | 0 | 72 (14.2) | 120 (23.6) | 144 (28.3) |
| 8. Stem cell transplantation can save lives. | 28 (5.5) | 0 | 72 (15.0) | 24 (4.7) | 380 (74.8) |
| 9. Stem cell therapy and cord blood harvesting are accepted in Islam. | 28 (5.5) | 0 | 72 (14.2) | 24 (4.7) | 384 (75.6) |
| 10. UCB units can still be used for transplantation years after they are first stored. | 72 (14.2) | 0 | 64 (12.6) | 60 (11.8) | 312 (61.4) |

Table 6: Association between mothers’ characteristics and their expectation level about UCB banking

| **Mothers’ characteristics** | **Total** | **Expectation score** | | **Significance** |
| --- | --- | --- | --- | --- |
|  |  | **Low** | **High** |  |
|  | **Number (Percent)** | | |  |
| Mother age in years  <25  25-30  31-35  >35 | 128 (100)  136 (100)  152 (100)  92 (100) | 120 (93.8)  124 (91.2)  104 (68.4)  92 (100) | 8 (6.2)  12 (8.8)  48 (31.6)  0 (0) | X=16.41  *P*=0.009 |
| Educational level  Illiterate  Primary education  Secondary education  University education | 24 (100)  76 (100)  264 (100)  144 (100) | 24 (100)  68 (89.5)  244 (92.4)  104 (72.2) | 0 (0)  8 (10.8)  20 (7.6)  40 (27.8) | X=10.24  *P*=0.036 |
| Occupation  Housewife  Working | 304 (100)  204 (100) | 280 (92.1)  160 (78.4) | 24 (7.9)  44 (21.6) | X=3.813  *P*=0.051 |
| Residence  Urban  Rural | 188 (100)  320 (100) | 128 (68.1)  312 (97.5) | 60 (31.9)  8 (2.5) | X=19.63  *P*<0.001 |
| Parity  Primipara  Multipara | 128 (100)  380 (100) | 96 (75.0)  344 (90.5) | 32 (25.0)  36 (9.5) | X=3.72  *P*=0.053 |
| Trimester  1st trimester  2nd trimester  3rd trimester | 132 (100)  200 (100)  176 (100) | 10 (90.9)  176 (88.0)  144 (81.8) | 12 (9.1)  24 (12.0)  32 (18.2) | X=1.48  *P*=0.477 |
| Family history of diseases  No history of diseases  Chronic diseases  Blood diseases | 216 (100)  196 (100)  108 (100) | 200 (92.6)  188 (95.9)  52 (54.2) | 16 (7.4)  8 (4.1)  44 (45.8) | X=27.12  *P*<0.001 |
| Total | 508 (100) | 440 (86.6) | 68 (13.4) |  |

P<.05 is significant
